# Supplementary material for: Disruption in daily eating-fasting and activity-rest cycles in Indian adolescents attending school
Source: PLoS One. 2020 Jan 10;15(1):e0227002. doi: 10.1371/journal.pone.0227002 (PMC6953840; doi:10.1371/journal.pone.0227002)
Supplement: Attachment — Submitted filename: Comments .docx [file pone.0227002.s001.docx]

The present manuscript has provided an insight for the daily lifestyles of young students and their feeding-fasting and rest activity cycles. I have few minor comments and questions regarding the present manuscript:

Minor comments:

1. The presents study did not explains about the male and female students in all the groups of the study? Please justify the number of male and females in each group.
2. And also provide the caloric differences between male and female group.
3. In figure 2C and 2D why there is high deviation in High school group weight and eating duration?
4. In result section under heading Daily eating patterns and body weight. You have written 651 days of recording. Is study was carried for approximately 2 years? If yes than it would be great if you can provide the change in caloric intake of each group in relation to seasonal changes.
5. In result section under heading Timing of daily caloric intake your paragraph second where you discuss about Fig 3. The labeling of figure 3 and results are not similar 3C is written twice in figure.
6. In result section under heading quantity of daily caloric intake why their is high FFc% among FY student? Do they have more preference to high sugar food or high fat food?
